# Functional assays reflective of cancer hallmarks in BT‐549 cells are not impacted by media supplemented with exercise‐trained plasma

**DOI:** 10.1113/EP091383

**Published:** 2023-11-22

**Authors:** Ian A. J. Darragh, Sarai Martinez‐Pacheco, Lorraine O'Driscoll, Brendan Egan

**Affiliations:** ^1^ School of Health and Human Performance Dublin City University Dublin Ireland; ^2^ School of Pharmacy and Pharmaceutical Sciences Trinity College Dublin Dublin Ireland; ^3^ Trinity Biomedical Sciences Institute Trinity College Dublin Dublin Ireland; ^4^ Trinity St. James's Cancer Institute Trinity College Dublin Dublin Ireland; ^5^ Florida Institute for Human and Machine Cognition Pensacola Florida USA

**Keywords:** breast cancer, exercise, metastasis, proliferation

## Abstract

Media supplemented with sera from acutely exercised men has been shown to have ‘anti‐cancer’ effects on prostate and breast cancer cell lines. This study investigated whether media supplemented with plasma samples taken at rest (≥30 h since the most recent exercise session) from men who were endurance‐trained (END), strength‐trained (STR) or recreationally active controls (CON) impacted the results of four assays that mimic hallmarks of cancer (proliferation, migration, extracellular matrix invasion and anoikis resistance) in the BT‐549 breast cancer cell line. Compared to control conditions of either serum‐free media or fetal bovine serum as appropriate, BT‐549 cells cultured with plasma‐supplemented media regardless of group resulted in greater cell proliferation (∼20–50%) and cell migration (∼15–20%), and lower extracellular matrix invasion (∼10–20%) and anoikis resistance (∼15–20%). Supplementing media with plasma from END or STR did not impact any outcomes of these assays compared to plasma from CON. Media supplemented with human plasma can impact functional assays reflective of cancer hallmarks in BT‐549 cells, but effects of exercise on proliferation, migration, extracellular matrix invasion and anoikis resistance were not evident in resting blood samples of individuals with a prior history of exercise training.

## Abstract (highlights)

- **What is the central question of this study?**
  Little is known regarding the effects of media supplemented with resting plasma from exercise‐trained individuals, despite the established bioactive effects of acutely exercised samples. Does media supplemented with resting plasma from endurance‐trained, strength‐trained or recreationally active controls impact hallmarks of cancer in BT‐549 cells?
- **What is the main finding and its importance?**
  Supplementing media with plasma from these trained athletes did not impact proliferation, migration, invasion or anoikis resistance compared to plasma from recreationally‐active controls. These findings suggest that ‘anti‐cancer’ effects of exercise are not present in resting blood samples of exercise‐trained individuals.

## 1 INTRODUCTION

Often referred to as an individual disease, ‘cancer’ describes a group of over 100 different diseases (Siegel et al., 2020; Weinberg, 1996), which share a common pathology described by an initial localized development, growth and proliferation of ‘abnormal’ cells followed by an eventual expansion to new tissues (Weinberg, 1996). There is phenotypic variability across cancer subtypes in terms of cell‐type origin, rate of occurrence, aggressiveness and responsiveness to treatment (Siegel et al., 2020). However, cancer cells have functional hallmarks, namely, sustained proliferative signalling; evasion of growth suppression; extracellular invasion and metastasis; replicative immortality; altered angiogenesis; and a resistance to cell death – which manifest to varying degrees across all cancer cell types (Hanahan & Weinberg, 2000, 2011).

A high level of habitual physical activity has been associated with decreased risk for incidence (relative risk reduction ∼10–20%), mortality (relative risk reduction ∼40–50%) and recurrence (relative risk reduction ∼25%) for several breast, prostate and colon cancers (Brown et al., 2012; Cormie et al., 2017; Mctiernan et al., 2019). There is currently limited understanding of the mechanisms by which physical activity or structured exercise training are preventative against cancer development, or provide remedial benefits to cancer patients. Commonly speculated mechanisms include increased tumour vulnerability mediated by alterations to tumour metabolism induced by acute exercise, and enhanced immune cell recognition of tumour cells, both of which may be caused by an exercise‐induced increase in circulating ‘exercise factors’ (e.g., Interleukin‐6) (Hojman et al., 2018; Metcalfe et al., 2021; Pedersen et al., 2016).

Given the associations between high levels of physical activity and reductions in cancer incidence, progression and mortality (Mctiernan et al., 2019), and the potential for exercise training to alter the resting profile of circulating factors (Chow et al., 2022), whether ‘anti‐cancer’ effects are present in blood samples from exercise‐trained individuals, that is, samples taken at rest and >24 h after the last exercise training session, is of increasing interest (Hojman et al., 2018; Metcalfe et al., 2021). *Ex vivo* cell culture models, where cells are cultured with media that is supplemented with human sera/plasma obtained from different physiological states (e.g., acutely exercised versus rested), are a feature in this domain (Allen et al., 2023; Metcalfe et al., 2021). This experimental model is intended to create cell culture conditions that are more representative of the circulating milieu, which may consequently elicit responses in cultured cells that are more translatable to in vivo human physiology (Allen et al., 2023).

In contrast to investigations examining sera/plasma from acutely exercised individuals (Metcalfe et al., 2021; Orange et al., 2020), investigations of the influence of sera/plasma from exercise‐trained individuals taken at rest on functional assays in cancer cells are quite limited (reviewed in Metcalfe et al., 2021). Therefore, the aim of this study was to investigate whether media supplemented with plasma from healthy men with divergent histories of exercise training impacted the results of four functional assays designed to mimic hallmarks of cancer (namely, proliferation, migration, extracellular matrix invasion and cell death/anoikis resistance) in the BT‐549 triple‐negative breast cancer‐derived cell line.

## 2 METHODS

### 2.1 Participants and study design

Men (*n* = 38) who were endurance‐trained (END; *n* = 13), strength‐trained (STR; *n* = 13) or recreationally active controls (CON; *n* = 12) were recruited for this study. Based on the recently proposed Participant Classification Framework (McKay et al., 2022), END comprised *n* = 8 tier 3/highly trained athletes and *n* = 5 tier 4/elite athletes, and STR comprised *n* = 9 tier 3/highly trained athletes and *n* = 4 tier 4/elite athletes. The phenotypic and performance characteristics of these participants are detailed extensively elsewhere (Darragh et al., 2023). Briefly, the STR participants’ recent one repetition maximum (1RM) in the squat exercise was 228 ± 30 kg, and the END participants’ recent 5‐km personal best was 16:30 ± 1:22 min:s. The CON participants were recreationally active (tier 2 of the Participant Classification Framework), but did not engage in any form of structured exercise training and self‐reported being unable to achieve a squat 1RM of >140 kg, or a 5‐km time of < 25:00 min:s. This study received ethical approval from the Research Ethics Committee of Dublin City University (DCUREC/2021/079) and conformed to the standards set by the *Declaration of Helsinki*, except for registration in a trials database. Each participant provided written informed consent prior to participation.

Participants were provided standardized meals (30 kcal/kg as 50/25/25% of energy intake for carbohydrate, protein and fat; GourmetFuel™, Dublin, Ireland) for the day prior to visiting the laboratory and were asked to abstain from alcohol consumption for at least 24 h. Specifically to mitigate any residual effects of recent acute exercise sessions (Darragh et al., 2021), participants were asked to refrain from exercise on the day before visiting the laboratory, which in practice resulted in all blood samples being taken at least 30 h after cessation of the last exercise session (CON, 81 ± 108 h; END, 45 ± 18 h; STR, 53 ± 48 h since last exercise session). Participants arrived after an overnight fast of at least 10 h and having only consumed water that morning. They lay supine for 10 min before blood sampling commenced with the insertion of a 21G butterfly needle (Greiner Bio‐One, Kremsmünster, Austria) into an antecubital vein. The initial 4 mL of whole blood was drawn into a generic vacutainer and discarded. Subsequently ∼50 mL of blood was drawn into six 9‐mL blood collection tubes coated with ACD‐A anticoagulant (Greiner Bio‐One, Austria). Blood samples were immediately placed on ice and later centrifuged at 1500 *g* for 15 min at 4°C. Directly after centrifugation, plasma samples were separated into aliquots, and stored at −80°C. Plasma samples were only defrosted once for use in the below‐described assays before being discarded.

### 2.2 Culturing of BT‐549 cells

BT‐549 cells (Lasfargues et al., 1978) were awoken from cryopreservation and cultured in T75 flasks containing RPMI 1640 medium (Moore et al., 1967) containing 10% fetal bovine serum (FBS), 5% l‐glutamate and 1% PenStrep. Cells were maintained in an incubator with a fixed temperature of 37°C and 5% CO_2_ and containing CuSO_4_. Cells were grown to ∼90% confluence and then either seeded or split. Cells were maintained for no more than 10 passages. All functional assay replicates used individual samples from CON, END and STR participants, with the individual participants used in each assay selected at random.

### 2.3 Sodium phosphatase cell viability (proliferation) assay

Cell proliferation was determined using an acid phosphatase assay that is based on the cellular conversion of *p*‐nitrophenol phosphate to *p*‐nitrophenol, the quantity of which correlates strongly with cell number (determined via haemocytometer counts), and is sensitive enough to detect as few as 1000 cells per well (Yang et al., 1996). BT‐549 cells were seeded in 96‐well plates at a concentration of 6 × 10^3^ cells per 100 µL, and to allow cells to fix to the plate, cells were cultured in their original media for 24 h. Next, cells were treated with 200 µL of one of the following 11 conditions: FBS‐free RPMI 1640 media (containing only 5% glutamate); FBS‐containing RPMI 1640 media (10% FBS, 5% glutamate); and RPMI 1640 media (containing 5% glutamate) supplemented with either 10, 15 or 20% human plasma from individual CON, END or STR participants. Each condition was applied to cells in quintuplicate, and to determine whether there was an influence of incubation period, replicates of cells were also incubated with these respective experimental conditions for periods of 24, 48 and 72 h.

On the day of each individual cytotoxicity assay, media was discarded from all wells, and cells were washed twice with 100 µL of room temperature phosphate‐buffered saline (PBS, Sigma‐Aldrich, cat. no.: P8537, St Louis, MO USA). Subsequently, 100‐µL solution of phosphatase substrate buffer consisting of 10 mM *p*‐nitrophenol phosphate diluted in a 1 M sodium acetate buffer (pH 5.5; 4.1 g of sodium acetate, Sigma‐Aldrich, cat. no.: S5636) diluted in 500 mL of dH_2_O and 500 µL of Triton X‐100 (Sigma‐Aldrich, cat. no.: T8787) was added to each experimental well. The plates were wrapped in aluminium foil and incubated with phosphatase substrate buffer for 1 h (at 37°C and 5% CO_2_). After incubation, the phosphatase reaction was stopped by adding 50 µL of 1 mM NaOH (VWR Chemicals, cat. no.: 27963.101, Dublin, Ireland), and absorbance was read at 405 nm using the FlouStar Optima microplate reader (BMG Labtech, Offenburg, Germany). Cell viability, as a marker of proliferation, was calculated by plotting absorbance values of each condition relative to the value obtained for the standard cell culture condition (RPMI media containing 10% FBS, 5% l‐glutamate and 1% PenStrep).

### 2.4 Cell migration assay

BT‐549 cells were seeded in a 24‐well plate at a concentration of 6 × 10^4^ per well in 500 µL of 10% FBS‐containing RPMI media for 24 h at 37°C and 5% CO_2_. After 24 h, cells were visually inspected to ensure proper formation of a cell monolayer. Each well was then ‘scratched’ by dragging a P200 pipette tip from the top to the bottom of the well in a single straight line (Liang et al., 2007). To remove floating dead cells, FBS‐containing media was discarded and each well was washed twice with 500 µL of media containing only RPMI media and 5% glutamate. Cells were then treated with fresh RPMI media that was modified to create the following five conditions: serum‐free media (SFM) containing only RPMI media, 5% glutamate, and 1% PenStrep; FBS comprising SFM with addition of 1% FBS; and three conditions comprising SFM with addition of 10% of human plasma from either a CON, END or STR participant. After treatment, each well was immediately visualized using a ×10 objective lens using an Olympus IX81 inverted microscope. This visualization constituted ‘0 h’ and at this time, a suitable region of the ‘wound’ was identified in each well and marked using a permanent marker. Cells were returned to an incubator and visualized again at this exact region at 24 h and 48 h post‐scratch. Cell migration was quantified in ImageJ using a bespoke plugin designed for wound‐healing assays (Suarez‐Arnedo et al., 2020).

### 2.5 Extracellular matrix invasion assay

Extracellular matrix (ECM) (Sigma‐Aldrich, cat. no.: E1270) diluted in SFM at a concentration of 1 mg/mL was thawed at 4°C. Two hundred microlitres of ECM was added to polyester transwell inserts with 8 µm pore sizes (Falcon, cat. no.: 353097, Corning Life Sciences, Fontainebleau, France) and left to incubate overnight (37°C and 5% CO_2_). The following day, BT‐549 cells were seeded to each insert at a concentration of 8 × 10^4^ cells in 200 µL of 10% FBS‐containing RPMI media. Cells were incubated in inserts for 24 h at 37°C and 5% CO_2_. FBS‐containing media was then removed from the inserts, which were washed twice with 200 µL of SFM, and then the inserts were treated with 200 µL of one of the following five conditions: SFM; SFM with addition of 1% FBS; and three conditions comprising SFM with addition of 10% of human plasma respectively from individual CON, END or STR participants. Five hundred microlitres of RPMI media containing 10% FBS was also added to the well surrounding each insert to promote cell invasion through the ECM. Cells were then incubated for 24 or 48 h at 37°C and 5% CO_2_. Post‐incubation, inserts were washed with a PBS‐soaked Q‐tip and incubated for 10 min with 0.1% crystal violet (diluted in deionized water) on a rocker at room temperature. Inserts were then washed again by gentle submersion in PBS, before being dried passively on paper. Visualization and imaging were performed using a ×10 objective lens of an Olympus IX81 inverted microscope to confirm ECM invasion. To quantify the quantity of invading cells, inserts were placed in 10% acetic acid (Sigma‐Aldrich, cat. no.: 338826) on a rocker for 10 min in order to elute the crystal violet. One hundred microlitres of eluate from each supplemented well was added to a 96‐well plate and the absorbance was read at 595 nm using a FluorStar Optima microplate reader. Cell invasion was quantified relative to the SFM‐treated control condition, which was assumed a priori to promote the greatest amount of invasion.

### 2.6 Anoikis resistance assay

To prevent cell adhesion, a 24‐well plate was coated with 200 µL of 12 mg/mL poly (2‐hydroxyethyl methacrylate) (poly‐HEMA; Sigma‐Aldrich, cat. no.: P3932) diluted in 95% ethanol, or a control condition of only 95% ethanol, and left in a fume hood overnight. This process was repeated twice, after which wells were seeded with BT‐549 cells at a concentration of 6 × 10^4^ per well in 500 µL of 10% FBS‐containing RPMI media. After seeding, wells were separated into five separate conditions by increasing the total amount of media in each well to a concentration of 600 µL through the addition of 100 µL of either serum‐free RPMI media (containing only 5% l‐glutamate; SFM condition); or 10% FBS; or human plasma taken from either CON, END or STR participants. Cells cultured in the 95% ethanol control (i.e., that could adhere to the plate) were cultured in 600 µL of 10% FBS. Cells were incubated in suspension for either 24 or 48 h, after wells were incubated with 60 µL of almarBlue dye for 3.5 h. Cell survival, as an index of anoikis resistance, was determined by reading plates at an absorbance of 570 nM on a FluoStar Optima microplate reader. Cell survival was quantified as relative to the 95% ethanol control (assumed to be 100% cell survival).

### 2.7 Statistical analysis

Data are reported or illustrated as means ± SD, where appropriate. Data were analysed in R using mixed linear models assembled with the ‘Rstatix’ package. A mixed model using the following formula for the initial proliferation experiment examined the main effects for Group (i.e., CON/END/STR or SFM/FBS controls), Plasma Dilution (10/15/20%) and Incubation Duration (24/48/72 h), and the interaction of these three main effects (Group × Plasma Dilution × Incubation Duration). Data from the remaining functional assay experiments (cell migration, ECM invasion and anoikis resistance) were analysed with models that examined main effects for Group and Incubation Duration (24/48 h), and their interaction (Group × Incubation Duration). When significant main or interaction effects were identified, differences were examined using *post hoc* comparisons with pairwise Student's *t*‐test. Results were considered statistically significant at an α‐level of <0.05. All models are reported with a relevant standardized effect size, namely partial eta squared (η_p_^2^), for which magnitudes of effect have been proposed to be interpreted at thresholds of small ≥0.01; moderate ≥0.06; and large ≥0.14 (Richardson, 2011).

## 3 RESULTS

### 3.1 Cell viability assay

**FIGURE 1:**
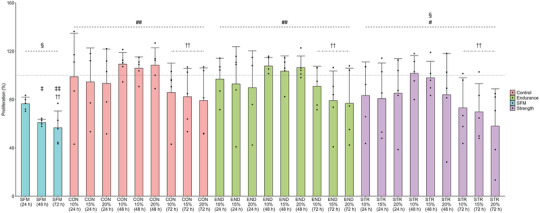
The results of a sodium phosphatase cell proliferation assay comparing cell culture media diluted with three concentrations of human plasma (10, 15 and 20%) from CON, END and STR participants across 24, 48 and 72 h (*n* = 5 individual samples for all groups). Bar height represents group mean and error bars represent standard deviation. †Significant difference from 48 h within groups; ‡significant difference from 24 h within groups; #significant difference from SFM group; §significant difference from FBS control (100% line) (all *P* < 0.05) – a single symbol represents *P* < 0.05, and a double symbol represents *P* < 0.01.

The results of a sodium phosphatase cell viability assay as an index of proliferation are presented in Figure 1. Significant main effects were observed for Group (*F* = 4.92, *P* < 0.01, η_p_^2^ = 0.08) and Incubation Duration (*F* = 13.2, *P* < 0.01, η_p_^2^ = 0.18) but no main effect was observed for the dilution percentage of human plasma (*F* = 1.21, *P* = 0.31, η_p_^2^ = 0.01) and no Group × Incubation Duration interaction (*F* = 0.136, *P* = 0.97, η_p_^2^ = 0.005) or Group × Plasma Dilution percentage was observed (*F* = 0.123, *P* = 0.97, η_p_^2^ = 0.004). *Post hoc* pairwise comparisons for Group indicated that dilution of RPMI media with plasma from CON (*P* < 0.01, mean difference: 24 h, 41%; 48 h, 46%; 72 h, 25%), END (*P* < 0.01, mean difference: 24 h, 16%; 48 h, 41%; 72 h, 25%) and STR (*P* < 0.05, mean difference: 24 h, 7%; 48 h, 33%; 72 h, 10%) had increased viability compared to SFM. The STR (*P* < 0.01, mean difference: 24 h, 16%; 48 h, 6%; 72 h, 33%) and SFM groups (*P* < 0.01, mean difference: 24 h, 23%; 48 h, 40%; 72 h, 43%) were also observed to have lower viability compared to the 10% FBS control. *Post hoc* pairwise comparisons on the main effect of Incubation Duration indicated that all human plasma groups had lower viability at 72 h compared to 48 h (*P* < 0.01, mean difference: CON, 25%; END, 24%; STR, 28%). The SFM group also had lower viability at 48 h (*P* = 0.01, 16% difference) and 72 h (*P* < 0.01, 20% difference) compared to 24 h.

### 3.2 Cell migration assay

**FIGURE 2:**
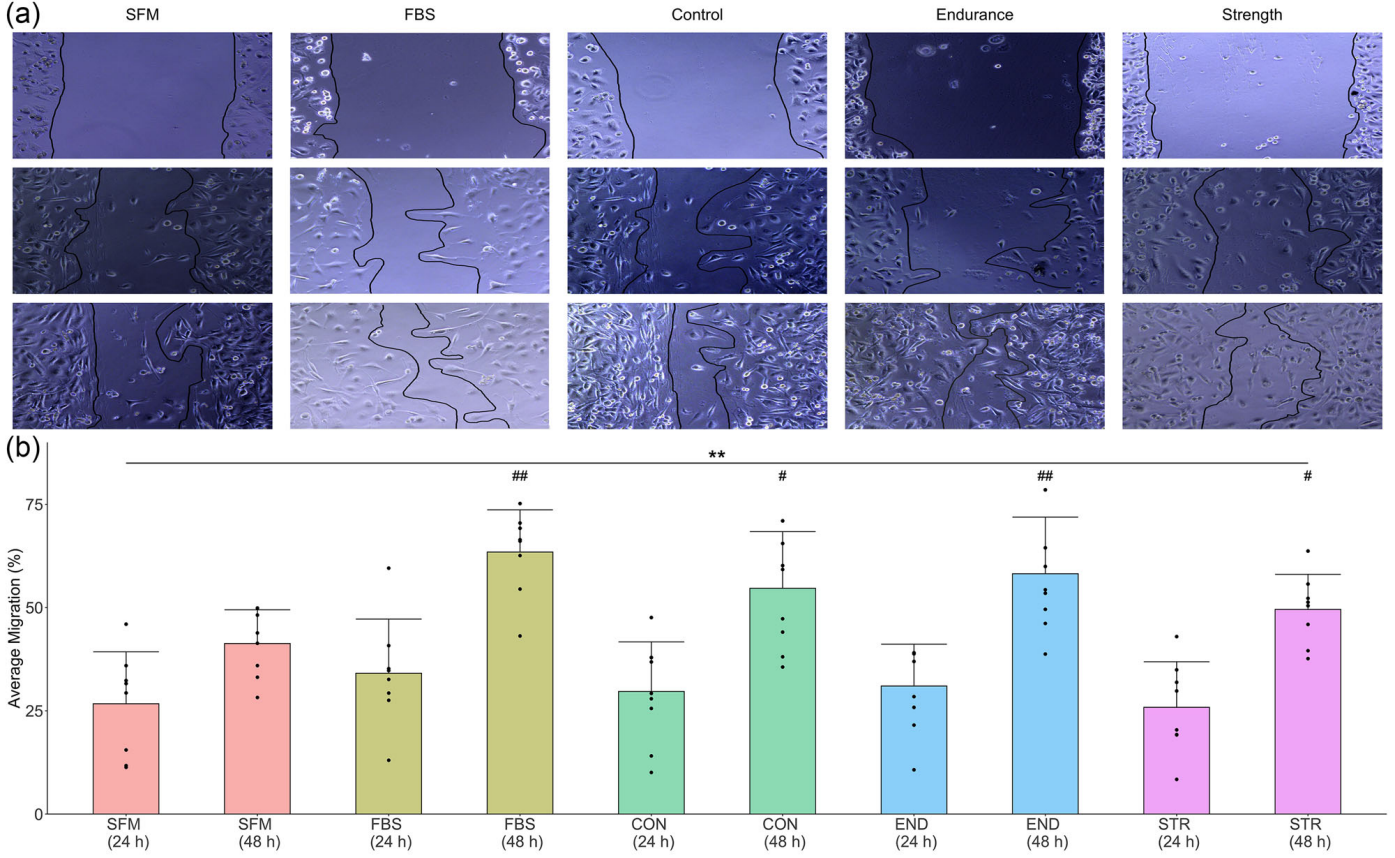
The results of a scratch assay promoting cell migration in BT‐549 cells (*n =* 7 individual samples for all groups). Cells were scratched and then incubated with SFM, 1% FBS‐containing RPMI or RPMI containing 10% of plasma from CON, END, or STR participants. Images were taken at 0, 24 and 48 h. Bar height represents group mean, and error bars represent standard deviation. (a) Representative images from each condition. (b) The average cell migration (change score from 0 h as a percentage) at 24 and 48 h. #Significant difference from SFM condition at 48 h; *significant difference between 24 and 48 h within groups – a single symbol represents *P* < 0.05, and a double symbol represents *P* < 0.01.

The results of a cell migration scratch assay are presented in Figure 2. Significant main effects were observed for Group (*F* = 4.05, *P* < 0.01, η_p_^2^ = 0.18) and Incubation Duration (*F* = 91.35, *P* < 0.01, η_p_^2^ = 0.55). No Group × Incubation Duration interaction effect was observed (*F* = 0.97, *P* = 0.42, η_p_^2^ = 0.05). *Post hoc* pairwise comparisons on the main effect of Group indicated a significant difference between all groups and the SFM condition at 48 h only (FBS: *P* < 0.01, mean difference: 22%; CON: *P* = 0.01, mean difference: 13%; END: *P* < 0.01, mean difference: 17%; STR: *P* = 0.03, mean difference: 8%). *Post hoc* pairwise comparisons on the main effect of Duration indicated a significant difference between 24 and 48 h (*P* < 0.01 mean difference: SFM, 15%; FBS, 30%; CON, 25%; END, 27%; STR, 24%). *Post hoc* pairwise comparisons on the main effect of Incubation Duration indicated a significant difference between 0 and 24 h (*P* < 0.01, mean difference: SFM, 37%; FBS, 52%; CON, 42%; END, 44%; STR, 35%) and 24 and 48 h (*P* < 0.01, mean difference: SFM, 25%; FBS, 79%; CON, 56%; END, 64%; STR, 47%).

### 3.3 Extracellular matrix invasion assay

**FIGURE 3:**
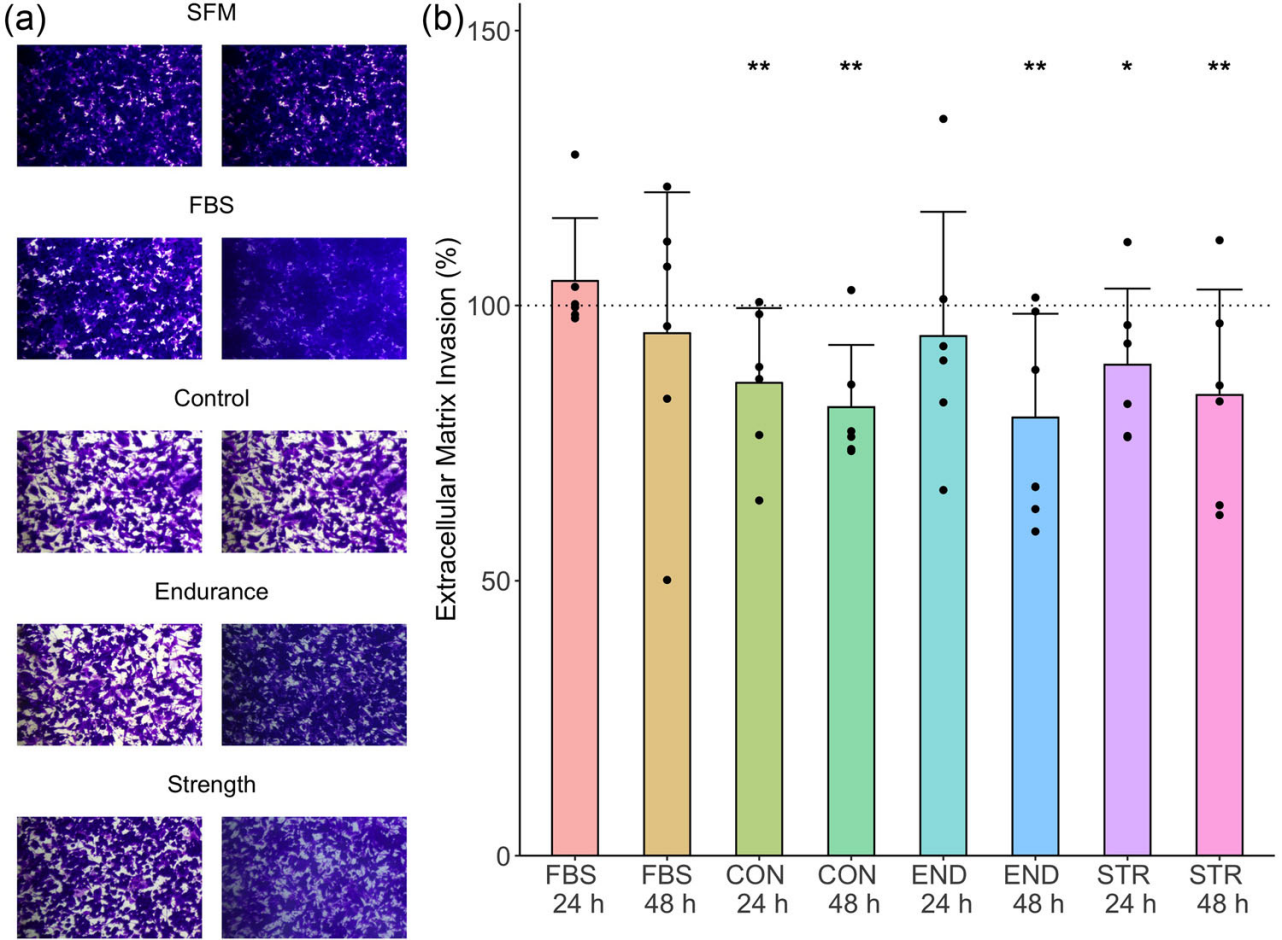
The results of extracellular matrix invasion assay in BT‐549 cells (*n =* 5 individual samples for all groups). Bar height represents group mean, and error bars represent standard deviation. (a) Representative images from each condition: left‐hand panel represents 24 h, and right‐hand side panel represents 48 h. (b) Results of four biological replicates of colorimetric quantification of matrix invasion. *Significant difference from the SFM condition, which is visualized as the 100% horizontal line – a single symbol represents *P* < 0.05, and a double symbol represents *P* < 0.01.

The results of an extracellular matrix invasion assay are presented in Figure 3. A main effect was observed for Group (*F* = 7.94, *P* < 0.01, η_p_^2^ = 0.27). No significant main effect was observed for Incubation Duration (*F* = 3.10, *P* = 0.08, η_p_^2^ = 0.03). *Post hoc* pairwise comparisons on the significant main effect for Group observed that the CON and STR groups both displayed significantly lower invasion than the SFM group at 24 h (CON *P* < 0.01, mean difference: 14%; STR *P* = 0.01, mean difference: 10%). CON, END and STR groups all had significantly lower invasion compared to the SFM control at 48 h (*P* < 0.01 for all groups, mean difference: CON, 18%; END, 20%; STR, 16%). No significant difference was observed between the 1% FBS group and SFM at either time point, and no significant differences between human plasma conditions were observed (all *P* > 0.05).

### 3.4 Anoikis resistance assay

**FIGURE 4:**
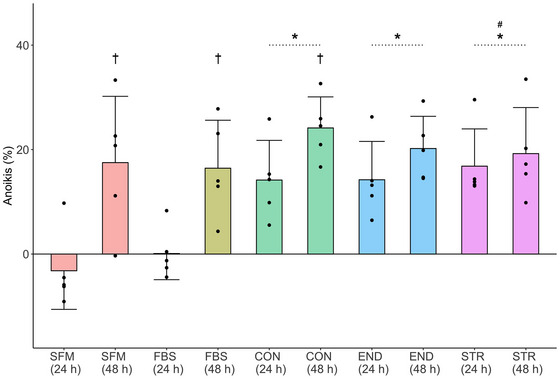
The results of a colorimetric anoikis resistance assay (*n* = 5 individual samples for all groups) in BT‐549 cells. Incubations were performed with media containing 15% plasma from CON, END and STR groups, 15% additional SFM, or 10% FBS RPMI. Bar height represents group mean and error bars represent standard deviation. *Significant difference from SFM condition; #significant difference from the 10% FBS condition; †significant difference between 24 and 48 h duration within a given group – all *P* < 0.05.

The results of an anoikis resistance assay are presented in Figure 4. A main effect was observed for Group (*F* = 5.2, *P* < 0.01, η_p_^2^ = 0.34) and Duration (*F* = 24.04, *P* < 0.01, η_p_^2^ = 0.36). No significant Group × Incubation Duration interaction was observed (*F* = 2.18, *P* = 0.08, η_p_^2^ = 0.18). *Post hoc* pairwise comparisons on the main effect of Group demonstrated significantly greater anoikis for all human plasma conditions compared to SFM (*P* < 0.01 for all groups, mean difference: CON, 17%; END, 17%; STR, 19%) and FBS (*P* < 0.01 for all groups, mean difference: CON, 14%; END, 14%; STR, 16%) at 24 h only. Anoikis increased significantly between 24 and 48 h for the SFM (*P* = 0.01, mean difference: 20%), FBS (*P* < 0.01, mean difference: 16%) and CON groups (*P* = 0.04, mean difference: 10%).

## 4 DISCUSSION

This study investigated whether culturing BT‐549 cells with media supplemented with plasma from healthy men with divergent histories of exercise training impacted assays for cell proliferation, migration, extracellular matrix invasion and anoikis resistance as functional hallmarks of cancer cells. Supplementing media with plasma from endurance‐ or strength‐trained athletes did not impact these assays compared to plasma from recreationally active controls, suggesting that anti‐cancer effects of exercise are not present in resting blood samples of individuals with a prior history of exercise training.

A salient issue for *ex vivo* cell culture models is determining the optimal concentration of sera/plasma to supplement media with and duration of cell exposure to supplemented media with an optimal concentration of sera/plasma speculated to vary depending on the cell line examined and outcome of interest (Allen et al., 2023). Previous work investigating media supplemented with human sera has used fixed concentrations of either 5 or 10% with incubation periods of 24–48 h (Metcalfe et al., 2021; Orange et al., 2020), which is consistent with *ex vivo* models applied to other cell types, namely skeletal muscle (Allen et al., 2023). Therefore, an important feature of the present study was establishing effects on the various assays in BT‐549 cells of media containing different concentrations of human plasma, regardless of training history, for a range of incubation periods. Cell proliferation was greater in all human plasma conditions (CON, END, STR) and incubation periods when compared to a SFM control, with the increases ranging from ∼20 to 50% in a manner that depended on the incubation duration, but not plasma concentration. Within each individual group (SFM, CON, END, STR), cell viability decreased between 48 and 72 h of incubation by ∼15–20% implying that this incubation period is excessive for optimal results on this assay. Considering these factors, we opted to use plasma concentrations of 10–15% and incubation periods up to 48 h for all subsequent experiments.

Interestingly, a prior history of exercise training had no influence on this proliferative effect, as evidenced by the lack of difference between END and STR compared to CON. These results contrast with previous work demonstrating that LNCaP cells incubated for 48 h with media supplemented with sera from men who participated in a community exercise programme (i.e., were purportedly ‘exercise‐trained’) had ∼40–60% lower cell proliferation compared to obese sedentary controls, as determined by cell‐counting on a haemocytometer (Tymchuk et al., 2001, 2002), or various colorimetric assays assessing proliferation (Barnard et al., 2007, 2003; Leung et al., 2004). However, all five of these studies utilized samples from the same cohort of participants and did not report the proximity to the most recent exercise training session for when the post‐training blood samples were taken (Barnard et al., 2007, 2003; Leung et al., 2004; Tymchuk et al., 2001, 2002). Proximity to the most recent exercise training session is an essential methodological consideration when assessing any biological outcome proposed as an effect of exercise training (Darragh et al., 2021; Egan & Sharples, 2023; Haskell, 1994). Notably in the present context, media supplemented with 5% sera from healthy men taken 0, 12 or 24 h after an acute exercise session decreases absolute cell counts of both LNCaP and MDA‐MB‐231 cells by ∼12–24%, whereas media supplemented with sera taken 72 h after exercise in the same participants had no influence on cell count number, even after 9 weeks of high‐intensity exercise training (Baldelli et al., 2021). Therefore, the decreased proliferation of LNCaP cells previously reported by other studies of ‘resting’ exercise‐trained (Barnard et al., 2007, 2003; Leung et al., 2004; Tymchuk et al., 2001, 2002) may not be an effect of exercise training per se because at 24 h after the last exercise training session, the residual influence of an acute exercise session cannot be entirely discounted (Darragh et al., 2021).

Using a scratch assay to assess cell migration, significant migration of BT‐549 cells was observed from 24 to 48 h in all conditions. Greater cell migration was observed in the 1% FBS condition and CON, END and STR plasma‐supplemented media conditions compared to the SFM control at 48 h, but again with no differences observed between the three human plasma conditions as an investigation of the effect of training history. In previous work, media supplemented with 1% of commercially acquired pooled human sera produced similar migration in both HeLa and SiHa cervical cancer cell lines compared to a 1% FBS condition (Heger et al., 2018). That study did not include a SFM control condition, but the observation that media supplemented with human sera promoted similar migration to a 1% FBS control is consistent with the present study.

With respect to the ECM invasion assay, media supplemented with 10% human plasma resulted in lower ECM invasion (by ∼10–20%) compared to the 10% FBS condition, but without differences between CON, END and STR. The reduction in invasion elicited by human plasma‐supplemented media was, therefore, also not influenced by the exercise training history of the participants. There is limited previous work investigating whether human sera/plasma‐supplemented media influences ECM invasion in cancer cells. One study investigated whether media supplemented with 10% of commercially acquired pooled human sera altered the ECM invasion of HeLa and SiHa cervical cancer cells, and observed that human sera increased invasion by ∼40 and ∼20%, respectively, compared to a 10% FBS control (Heger et al., 2018).

An important methodological difference is that in the present study, 10% human plasma was added to the ECM‐containing transwell 24 h after cells were seeded, after which 10% FBS‐containing media was placed around the transwell to encourage cells to migrate through the ECM. In contrast, the previous study seeded all cells in SFM, and instead investigated whether adding 10% human sera‐supplemented media around the transwell would alter invasion in comparison to 10% FBS (Heger et al., 2018). We chose to add plasma directly to the cells (i.e., *within*, instead of around, the transwell) in order to investigate whether culture of BT‐549 cells with media supplemented with human plasma would alter BT‐549 invasion through the ECM, rather than investigating whether human sera acted as a stronger signal for cell migration compared to 10% FBS (Heger et al., 2018). However, our experimental set‐up makes it difficult to infer whether incubation with media supplemented with 10% human plasma reduced the function of BT‐549 cells to a lower capacity for ECM invasion (i.e., an ‘anti‐cancer’ effect), or whether the supplemented media was sufficiently ‘nourishing’ (as also arguably evidenced by the results of the proliferation assays above) for BT‐549 cells to reduce the stimulus for cells to migrate through the ECM. Further and more detailed experimentation is required to determine which situation is more likely to be the case with respect to this assay.

Anoikis is a form of programmed cell death that is initiated when cells become detached or inappropriately attached to their relevant ECM, such that resistance to anoikis is essential for tumour metastasis (Taddei et al., 2012). BT‐549 cells are derived from the epithelial cells of a metastatic ductal tumour and therefore possess anoikis resistance (Lasfargues et al., 1978). The present study observed that suspended BT‐549 cells that had their culture media supplemented with 15% human plasma had higher rates of anoikis (i.e., reduced anoikis resistance) of ∼14–19% compared to cells treated with SFM or 10% FBS after 24 h of incubation, but not after 48 h. Again, there was no evidence of an influence of training history when comparing CON, END and STR. Therefore, media supplemented with human plasma may have only accelerated an inevitable induction of anoikis in these cells. Anoikis will occur in control conditions in BT‐549 cells, with activation of cleaved caspase 3, 8 and 9, and anoikis values of ∼8–12% have been observed in suspended BT‐549 cells after 12.5 h incubation in 2% FBS in RPMI media (Zheng et al., 2020). The delay in anoikis in the FBS condition in the present study may be due to the greater amount of FBS used (10%), particularly as the parameter of the SFM control was the *addition* of 100 µL of SFM to wells already containing media that was enriched with 10% FBS.

Why media supplemented with human plasma would cause an earlier induction of anoikis remains to be explained, particularly when the results of the cell proliferation and migration assays, which suggested a ‘nourishing’ effect of human plasma, are considered. No study has previously investigated the influence of media supplemented with human sera/plasma on anoikis resistance. However, three studies have reported that incubation with media supplemented with 10% human sera for 48 h induces apoptosis in LNCaP cells (Barnard et al., 2007, 2003; Leung et al., 2004), with increased p53 protein expression also observed (Leung et al., 2004). In contrast, 72 h of incubation with media supplemented with 10% resting and acutely exercised sera was reported not to induce apoptosis in CaCo2 or LoVO cells (Devin et al., 2019). Therefore, there is uncertainty with respect to how robust the influence is of media supplemented with human plasma on anoikis and other pathways of cell death, or by which mechanisms human plasma may induce acceleration of cell death in cancer cell lines.

Given that the influence of sera/plasma from acutely exercised individuals appears robust (Metcalfe et al., 2021; Soares et al., 2021), the extent to which inferences can be made with respect to how exercise training history influences the effects of human plasma‐supplemented media are limited in the present study by the absence of an acute exercise condition as a ‘positive’ control. While no divergent effects between CON, END and STR groups were observed, generic effects of using media supplemented with human plasma were observed across all four functional assays. In some circumstances, these produced results that were clearly ‘beneficial’ to BT‐549 cells, that is, the proliferation and migration assays, but for the ECM invasion and anoikis resistance assays, results were less clear regarding whether the supplemented media had negligible, beneficial or deleterious effects on BT‐549 cells. This study is also limited by the selection of only one cell line, which reduces the generalizability of findings on the influence of supplemented media to other types of cancer cells.

There are several limitations to the present study that must be acknowledged. First, this study only investigated one cell line (BT‐549 cells) from one type of cancer (triple‐negative breast cancer), so including additional cell lines would increase the robustness and generalizability of our findings. Second, we inferred the fitness of participants from performance data (race performance for END participants, and 1RM values for STR participants), but did not perform laboratory measurement of physical fitness in our participant (e.g., measurement of maximal oxygen uptake as ${\dot{V}}_{O_{2}\max}$). Third, the CON participants were recreationally active, which was the experimental design a priori because our specific research question pertained to whether a prior history of exercise *training* influenced the response of BT‐549 cells to each functional assay. Using control participants who were sedentary may have introduced confounding effects due to mild metabolic dysfunction (Booth & Lees, 2006; Booth et al., 2017), but it is possible that such an approach would have increased the likelihood of observing differences between groups. Lastly, replicates of *n* = 5 or 7 were used in the functional assays, and therefore it is possible that an insufficient number of replicates was used and that this increased the likelihood of failing to detect differences between CON, END and STR participants, that is, a type II error.

In conclusion, while media supplemented with human plasma impacted functional assays in BT‐549 cells across all four assays reflective of cancer hallmarks, no differences between CON, END or STR groups were observed in this experimental model. These data suggest that exercise training history does not influence the effects of media supplemented with human plasma when blood samples are taken at rest, at least when participants are otherwise healthy. The contention is, therefore, that the anti‐cancer effects of exercise are not present in resting blood samples of individuals with a prior history of exercise training, and any beneficial effects are largely conferred by the effects of acute exercise sessions in the overall context of an exercise training programme.

## AUTHOR CONTRIBUTIONS

Ian Darragh: conceptualization; methodology; formal analysis; investigation; writing—original draft. Sarai M. Pacheco: methodology; writing—review and editing. Lorraine O'Driscoll: writing—review and editing; supervision; funding acquisition. Brendan Egan: conceptualization; methodology; writing—review and editing; supervision; project administration; funding acquisition. All authors have read and approved the final version of this manuscript and agree to be accountable for all aspects of the work in ensuring that questions related to the accuracy or integrity of any part of the work are appropriately investigated and resolved. All persons designated as authors qualify for authorship, and all those who qualify for authorship are listed.

## CONFLICT OF INTEREST

The authors have no conflict of interests, financial or otherwise, to declare.

## Data Availability

Data are available via request to the corresponding author
